# Correlations between Steady-State Pattern Electroretinogram and Humphrey Visual Field Analyzer Global Indices and Their Associations with Retinal Ganglion Cell Layer-Inner Plexiform Layer Thickness in Glaucoma Suspects

**DOI:** 10.1155/2024/2443887

**Published:** 2024-03-11

**Authors:** Andrew Tirsi, Vasiliki Gliagias, Daniel Zhu, Benny Wong, Rohun Gupta, Sung Chul Park, Stephen Obstbaum, Celso Tello

**Affiliations:** ^1^Manhattan Eye, Ear and Throat Hospital, New York, NY, USA; ^2^Donald and Barbara Zucker School of Medicine at Hofstra University/Northwell Health, New Hyde Park, NY, USA; ^3^Northwell Health, New Hyde Park, NY, USA

## Abstract

**Purpose:**

The purpose of this study was to investigate the utility of steady state pattern electroretinogram (ss-PERG) in detecting retinal ganglion cell (RGC) dysfunction in glaucoma suspects (GS) who had normal 24-2 Humphrey Visual Fields (HFA).

**Materials and Methods:**

This was a prospective cohort study of GS patients who were identified based on optic disc appearance with normal HFAs. Patients received a complete eye examination, standard automated perimetry (SAP), optical coherence tomography (OCT), and ss-PERG measurements. The ss-PERG parameters, Magnitude (Mag), Magnitude D (MagD), and MagD/Mag ratio, were examined, along with their relationships between HFA and OCT measurements.

**Results:**

Twenty-five patients were included in this study, with a total of 49 eyes. Fifteen eyes had abnormal ss-PERG parameters and when compared to GS eyes with normal ss-PERG parameters, there were significant differences in HFA 24-2, retinal nerve fiber layer (RNFL) thickness, and ganglion cell layer and inner plexiform layer (GCL + IPL) thickness. All ss-PERG parameters were significantly correlated with 24-2 VF mean deviation (MD) and visual field index (VFI), as well as 10-2 VF MD after controlling for age, sex, intraocular pressure, central corneal thickness, and spherical equivalent. When controlled for age, spherical equivalent, and IOP, MagD/Mag ratio significantly contributed to the variance in average GCL + IPL thicknesses, whereas 24-2 VF MD and 10-2 VF MD did not. MagD/Mag ratio also significantly accounted for variance in all macular GCL + IPL sectors, while 10-2 VF MD did not.

**Conclusions:**

ss-PERG has significant correlations with HFA global indices and was predictive of GCL + IPL thickness in GS patients. *Clinical Significance*. ss-PERG may serve as a useful functional tool for detecting and measuring RGC dysfunction in GS. It appears to be more sensitive than HFA in the detection of early changes in GCL + IPL thicknesses and may be helpful to use in conjunction with current diagnostic studies to improve the ability of monitoring GS progression.

## 1. Introduction

Glaucoma is a neurodegenerative disease of the optic nerve characterized by chronic damage to retinal ganglion cells (RGC), which leads to irreversible visual impairments if not adequately treated. Typically, the disease progresses gradually and remains asymptomatic until a significant amount of visual function is lost, which is detected by standard automated perimetry (SAP).

The definition of a glaucoma suspect (GS) is a person with one or more clinical features and/or risk factors for glaucoma. The clinical characteristics can include elevated intraocular pressure (IOP), optic nerve head or retinal nerve fiber layer (RNFL) appearance suggestive of glaucomatous damage, unexplained visual field (VF) defect consistent with glaucoma, abnormal angles or strong family history of severe glaucoma, and other risk factors [1]. A histological study on glaucomatous eyes demonstrated that early abnormalities in SAP were detected when at least 25% to 35% of RGCs were lost [2, 3], suggesting that patients with early glaucoma already had significant cell injury [4]. In addition, optical coherence tomography (OCT) studies reported that RNFL loss was observed in 60% of eyes approximately 6 years before the threshold of glaucoma detection was reached and its characteristic VF loss was discovered in SAP [2, 5]. Furthermore, while structural evaluations of glaucoma rely on objective methods such as OCT, functional evaluations of the disease still rely on subjective VF assessment. Therefore, an objective functional test to detect early RGC dysfunction in GS would be useful in the prevention of decreased quality of life and falls [6, 7].

Pattern electroretinogram (PERG) is an electrophysiological test that uses stimuli consisting of temporally phase-reversed black and white horizontal/checkerboard gratings. The contrast and viewing angle of the stimulus are optimized to elicit detection of RGC dysfunction selectively. The origin of PERG signal has been demonstrated in animal studies in 1981, when Maffei and Fiorentini recorded standard flash electroretinogram (ERG) and PERG signals from the cat retina and then sectioned its optic nerve, triggering retrograde RGC death. They observed an abolishment of the PERG signal, while the standard flash ERG signal, known to be generated in the outer retina, remained intact [8]. Subsequently, more studies confirmed similar findings on animals by inflicting a temporary retinal ischemia by either clamping of the retinal artery or by increasing IOP, leading to a decrease in PERG amplitude but no effects on flash ERG [8, 9]. Histological examination of whole-mounted animal retinas showed loss of RGC and intact outer retina neurons, demonstrating the importance of RGC integrity for the generation of a normal PERG response [10].

Therefore, PERG is a proven and accepted clinical tool in the assessment of the macula and RGC function [11–15]. Previous studies have demonstrated RGC dysfunction and PERG alteration in early glaucoma [16], ocular hypertension, and experimental models of glaucoma [17]. Furthermore, PERG studies were able to detect early RGC dysfunction in glaucoma 4 years before VF defects occurred [12, 16]. PERG testing provides an objective measure of central retinal function to discriminate between normal and early glaucoma subjects [15, 18]. The PERG stimulus can be based on contrast variation of either low (transient response) or high (steady state response) temporal frequencies; fast steady state stimulus has been shown to have better glaucomatous dysfunction than transient slow stimulus, and this phenomenon has been explained by the RGCs having been submitted to a greater metabolic stress [19–21].

In one study including early glaucomatous eyes with relative and absolute scotomas, authors concluded that glaucoma starts with subclinical pan retinal damage of the RGCs, reflected in the ss-PERG test results, but not necessarily in conventional VF examination [22]. We have previously studied ss-PERG in GS and found that ss-PERG parameters were associated with RNFL thickness [23].

The primary purpose of this study was to ascertain whether ss-PERG testing had the ability to detect RGC dysfunction in GS eyes with normal Humphrey Field Analyzer (HFA) 24-2 Swedish Interactive Threshold Algorithm (SITA)-standard test. Our secondary purpose was to correlate ss-PERG parameters with HFA 10-2 global indices to determine the clinical significance of their relationships. Lastly, we sought to determine which functional test had a stronger association with spectral-domain (SD)-OCT derived retinal ganglion cell layer-inner plexiform layer (GCL + IPL) thickness measurements in GS.

## 2. Materials and Methods

All subjects were recruited from the Manhattan Eye, Ear, and Throat Hospital between March and September 2017 and underwent a complete ophthalmologic examination, including slit lamp biomicroscopy, Goldmann applanation tonometry, central corneal thickness (CCT) measurement (PachPen®, Accutome, Inc., Pennsylvania, USA), standard automated perimetry (Humphrey Field Analyzer II, 24-2 and 10-2 SITA-Standard strategy, Carl Zeiss Meditec Inc., Dublin, CA, USA), SD-OCT (Cirrus ® HD-OCT, Carl Zeiss Meditec Inc., Dublin, CA, USA), and ss-PERG NOVA® (Diopsys Inc., Pine Brook, NJ, USA). The study was approved by the Institutional Review Board of Northwell Health System. Written informed consent was obtained from all subjects, and the study adhered to the tenets of the Declaration of Helsinki.

We followed the methods of Andrew Tirsi et al. [23–26]. GS participants were recruited according to the following criteria: the presence of a glaucomatous optic nerve head appearance (cup-to-disc ratio asymmetry of >0.2 between fellow eyes, neuroretinal rim thinning, notching, or excavation) and a normal HFA 24-2 SITA-standard test. Participants of ages 20–80 years, best corrected visual acuity better or equal to 20/40, spherical refraction within ±6.0 D, and cylinder correction within 3.0 D were included. Using the HFA 24-2 SITA-standard test, only participants with stage 0 (no visual field losses), based on the Glaucoma Staging System (GSS 2), were enrolled in this study [27]. Participants had no history of treatment with IOP-lowering drops. Using the Glaucoma Hemifield Test (GHT), the normal HFA test was defined (pattern standard deviation (PSD) within 95% confidence limits and mean deviation (MD) ≥−2 dB). Individuals with unreliable HFA results, fixation losses, and false positive or negative rate >20% were excluded. Prior intraocular surgery (except for uncomplicated cataract extraction), ocular trauma, and ocular or systemic conditions that may affect the optic nerve head or retinal structure or function (e.g., ischemic optic neuropathy, optic neuritis, papilledema, and retinal diseases) resulted in exclusion. Juvenile open angle glaucoma, primary developmental glaucoma, pigment dispersion syndrome, and pigmentary glaucoma were excluded from this study.

OCT scans of the macula were obtained using Cirrus ® HD-OCT (software version 9.0.0.281) as described previously [28]. Ganglion cell analysis was generated and displayed as sectorial (superior, superior nasal, inferior nasal, inferior, inferior temporal, and superior temporal sectors) thickness and as average GCL + IPL (aGCL + IPL) thickness. Images with signal strength <6 or with visible eye motion or blinking artifacts or algorithm segmentation failure were discarded.

### 2.1. Steady State Pattern Electroretinogram (ss-PERG)

Ss-PERGwas recorded using a commercially available system, Diopsys® NOVA-ss-PERG, and the methodology was described in previous works [23–26]. In summary, a total of 3 electrodes were used per test for each patient (two active/reference and one ground electrodes). Subjects were fitted with the appropriate correction for a viewing distance of 24 inches and were instructed to fixate on a target at the center of the monitor. No pupil dilation was performed. If more than 4 artifacts were recorded over one 25-second period, subjects were instructed to reduce blinking frequency, and eye lubricants were offered if needed.

The pattern stimulus consisted of black/white square-wave horizontal gratings (grating size 64 × 64, 24°, 100% contrast and 102.4 candelas/m^2^ mean luminance), reversing at 15 reversals/second (rps) with a duration of 25 seconds for high contrast [Hc 85%] and 25 seconds for low contrast [LC 15%] for a total of 50 seconds per eye. An automatic discrete Fourier transformation (FFT) was applied to the PERG waveforms to isolate the desired component at 15 rps. Ss-PERG test results were saved in a structured query language (SQL) database and presented in a report form to be used for further statistical analysis. The device collected 5 frames of data per second, totaling 125 frames of data, and the first 10 frames (2 seconds) of data were discarded. A result was categorized as nonreliable if there were more than 4 artifacts [23–26].

Five ss-PERG measurements (Magnitude, MagnitudeD, MagD/Mag ratio, number of artifacts, and signal to noise ratio (SNR) per test) for each eye were collected. Magnitude (Mag, in *µ*V) indicated the amplitude or the signal strength at the specific reversal rate of 15 Hz, in the frequency domain. MagnitudeD (MagD) represented the amplitude of the ss-PERG signal and its relation to phase variability throughout the waveform recording. MagD/Mag ratio is a ratio that is within-subject representation of the phase consistency and denotes retinal signal with intrinsic variability [23–26].

### 2.2. Statistical Analysis

For all variables of interest, outliers with values ≥3 standard deviations from the mean were excluded from the analyses. The Shapiro–Wilk test was used to determine normality of the distribution for all important variables. All abnormal distributed variables of interest were therefore subjected to either logarithmic transformations or to a 2-step fractional ranking approach described elsewhere [29]. All transformed continuous variables were subsequently used in correlation and regression analyses. Descriptive statistics were used to evaluate continuous and demographic data. Mean and standard deviation values were determined for each ss-PERG (Mag, MagD, MagD/Mag ratio), HFA SITA-Standard (24-2 and 10-2) tests, and all sectorial and average GCL + IPL thickness variables.

Using MagD/Mag ratio as a measure of RGC function, we created a control group with normal RGC function (MagD/Mag ratio ≥0.752) and a second group with objective RGC dysfunction (MagD/Mag ratio <0.752). Differences between the two groups were analyzed using independent *t*-tests and chi-square tests of independence.

Pearson correlation coefficients were used to test the correlation between ss-PERG measurements and HFA parameters. To better understand whether there is a linear relationship between ss-PERG and HFA parameters, while controlling for risk factors for glaucoma, such as age, sex, CCT, spherical equivalent, and IOP, we performed partial correlation analysis.

Generalized linear mixed modeling (GLMM) was used to further assess the relationships among ss-PERG, OCT, and HFA parameters. All GLMMs utilized an unstructured covariance model with a randomly generated intercept. The GLMMs analyzed each patient with measures of each eye classified as repeated measures to minimize within-subject intereye correlations. Postestimation analysis was conducted using the Satterthwaite approximation due to the utilization of an unstructured covariance model.

Because GCL + IPL parameters have comparable diagnostic abilities as circumferential RNFL for early glaucoma [30], it was of interest to use hierarchal multiple regression analyses to predict future GCL + IPL thickness variance. Independent variables that were significant in exploratory stepwise regression analyses or conceptually important variables based on our review of the literature were included in the final regression analyses. To predict average GCL + IPL thickness change, we adjusted for age, sex, CCT, IOP, and spherical equivalent in the first step, MD 24-2 in the 2^nd^ step, and MagD/Mag ratio in the last step. Subsequently, we used a similar model predicting average GCL + IPL by replacing MD 24-2 with MD 10-2. Finally, we used identical regression models to predict future sectorial GCL + IPL thickness change by replacing average GCL + IPL thickness by sectorial GCL + IPL thickness (superior temporal, superior, superior nasal, inferior nasal, inferior, and inferior temporal) each time, keeping the same predictors. Statistical analyses were performed with commercially available software (IBM® SPSS® ver.23.0; SPSS Inc, Chicago, IL, USA).

## 3. Results

Fifty-four eyes (2 GS patients) were initially recruited. Three eyes of 2 patients were excluded due to poor quality OCT scans, and 2 eyes of 1 patient were excluded due to abnormal or unreliable VF results. A total of 49 eyes (25 patients) were included in the analysis. The characteristics of the study population are summarized in Table 1. The mean age was 58.96 years, and 16 participants were females (64%). The baseline mean HFA MD 24-2 was −0.0004 dB, and the mean IOP was 17.43 mmHg.

### 3.1. Detection of Early RGC Dysfunction in GS

Fifteen eyes exhibited RGC dysfunction by decreased MagD/Mag ratio (*R* < 0.752, borderline and outside reference range), among which 8 eyes exhibited decreased MagD (MagD <0.752, borderline and outside reference range) and 4 eyes decreased Mag (Mag <1.00, borderline and outside reference range). There was a significant difference between eyes with normal ss-PERG and those with abnormal ss-PERG in age, 24-2 VF MD, 24-2 VF PSD, and VFI. GS eyes with abnormal ss-PERG (MagD/Mag ratio <0.752) also exhibited significantly thinner circumpapillary RNFL globally (85.50 ± 9.66 vs 92.75 ± 9.04 *µ*m), in the superior quadrant (95.07 ± 13.46 vs 110.22 ± 17.58 *µ*m) and in the inferior quadrant (109.64 ± 15.79 vs 122.12 ± 12.38 *µ*m), as well as a significantly smaller rim area (1.02 ± 0.15 vs 1.18 ± 0.17 mm^2^), minimum macular GCL + IPL thickness, and macular GCL + IPL thickness in the superior sector and in the superior temporal sector *p* < 0.041) (Table 2).

### 3.2. Relationships between ss-PERG and HFA Global Indices

Bivariate Pearson correlation revealed significant associations between VF MD (both 24-2 and 10-2) and all ss-PERG parameters (*r* > 0.383, *p* < 0.009). There was no significant correlation between ss-PERG and 10-2 VF PSD, whereas 24-2 VF PSD was significantly correlated with MagD/Mag ratio (*r* = 0.357, *p*=0.013) (Table 3).

**Figure 1:**
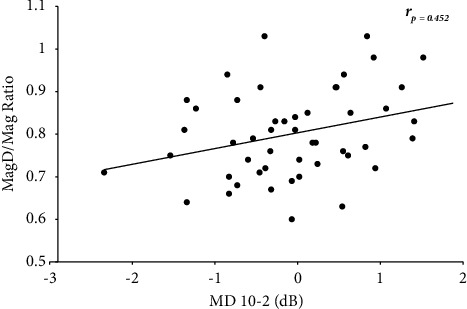
Scatterplot of the relationship between MagD/Mag ratio and 10-2 VF MD values after controlling for age, sex, CCT, SE, and IOP (*r*_*p*_ = 0.452; *p* < 0.003). MagD/Mag ratio: MagnitudeD/Magnitude ratio, MD: mean deviation, dB: decibels, and rp: partial correlation.

Partial correlation analyses were performed to determine the relationship between HFA global indices and ss-PERG parameters after controlling for known glaucoma risk factors such as age, sex, IOP, CCT, and spherical equivalent. [29–31] All ss-PERG parameters were positively correlated with 24-2 VF MD (*r* > 0.390, *p* < 0.012), 10-2 VF MD (*r* > 0.452, *p* < 0.003), and VFI (*r* > 0.419, *p* < 0.006) (Table 3). Scatterplot results representing the relationship between MagD/Mag ratio and MD 10-2 HF MD are shown in Figure 1.

### 3.3. ss-PERG, HFA MD, and Their Associations with aGCL + IPL Thickness

**Figure 2:**
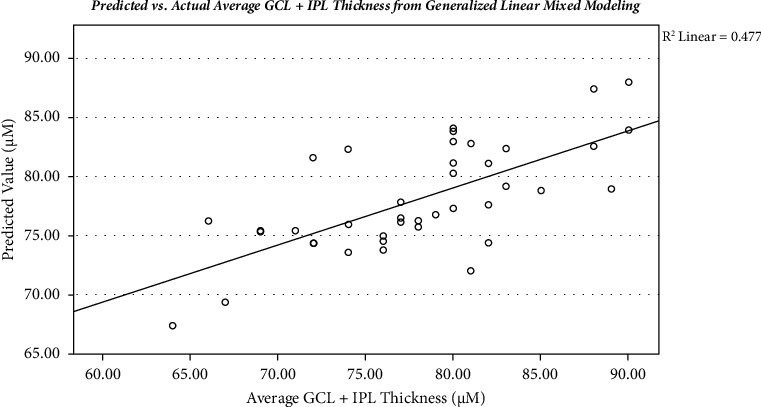
Scatterplot of the relationship between predicted and actual average GCL + IPL thickness from generalized linear mixed modeling (*r*_*p*_ *=* 0.477; *p* < 0.003). GCL + IPL: ganglion cell inner plexiform layer.

To minimize the effect of within-subject intereye correlations, a GLMM with both eyes in each subject classified as a repeated measure was used to predict average GCL + IPL thickness after utilizing age, IOP, spherical equivalent, and utilizing MagD/Mag ratio as fixed measures. The GLMM yielded a significant equation (*R*^2^ = 0.474, *p*=0.002) with each fixed variable significantly contributing to the overall variance (Table 4) (Figure 2). MagD/Mag ratio explains 9.47% of the variance, whereas age, IOP, and spherical equivalent all contributed to <1% of the overall variance. Predicted values from the GLMM correlated significantly with average GCL + IPL thickness values (*R* = 0.690, *p*=0.002) (Table 4). Participant's average GCL + IPL thickness decreased by 26.8 *μ*m for each unit decrease of the MagD/Mag ratio.

In parallel analysis, GLMMs were applied to sectorial GCL + IPL (superior, superior nasal, inferior nasal, inferior, inferior temporal, and superior temporal) thicknesses, with age, IOP, spherical equivalent, and MagD/Mag ratio as fixed prediction variables. All GLMMs yielded significant equations (*p* < 0.003) (Table 5) predicted in superior, superior nasal, inferior nasal, inferior, inferior temporal, and superior temporal GCL + IPL sectors. Sectorial GCL + IPL thickness analyses (sectors S, SN, IN, I, IT, and ST) revealed that for each unit decrease of the R, sectorial thickness decreased by 33.1 *µ*m, 29.3 *µ*m, 23.7 *µ*m, 24.4 *µ*m, 20.6 *µ*m, and 24.1 *µ*m, respectively. 10-2 VF MD did not contribute any variance.

## 4. Discussion

In this study, we demonstrated that GS (15 eyes) presented with abnormal ss-PERG test results suggesting the presence of early RGC dysfunction. While glaucoma is clinically defined as optic nerve head change with corresponding VF defects, the pathophysiology of the disease lies in the ultimate loss of RGC [32]. Early detected abnormalities in SAP were significantly associated with 25% to 35% of RGC loss, indicating significant cell injury in the early stages of the disease [2, 3]. It has been demonstrated that RGC loss occurs approximately 6 years before its characteristic visual field detection suggesting that RGC dysfunction might already be present in GS [4], and this study's results confirm those findings.

Accumulating evidence from experimental and clinical studies suggests that RGCs in their earliest stages of glaucoma can recover their function following periods of their dysfunction [33]. A study by Crowston et al. described the mechanism of RGC damage in glaucoma [34]. As a response to injury, Crowston et al. suggested that RGCs will undergo repair, and ideally, functional loss will be followed by full recovery. A subpopulation of cells will undergo cycles of injuries and repairs [34]. When the RGCs are no longer able to repair, they surpass a threshold and a cell death program is initiated leading to apoptosis and structural damage [34]. Meanwhile, if RGC dysfunction happens first, it is still reversible in the early stages of disease. However, structural damage follows if the cells fail to repair in response to IOP elevation or other metabolic and vascular challenges. These homeostatic thresholds are believed to vary across ganglion cells and between individuals, as well as with age, stage of disease, and comorbidities. Glaucoma has been traditionally viewed as a disease of the optic nerve in which RGC axons sustain the initial insult, followed by degeneration of stroma [35]. Morphologic analysis of RGC in glaucoma animal models has shown that axons are affected first and then their cell bodies, at different times after an IOP increase [36]. Morquette et al. suggested that atrophy of RGC dendrites precedes cell body shrinkage and axonal breakdown in glaucoma, concluding that dendritic abnormalities and synaptic loss may be an early feature of vision loss in glaucoma [37–39].

**Figure 3:**
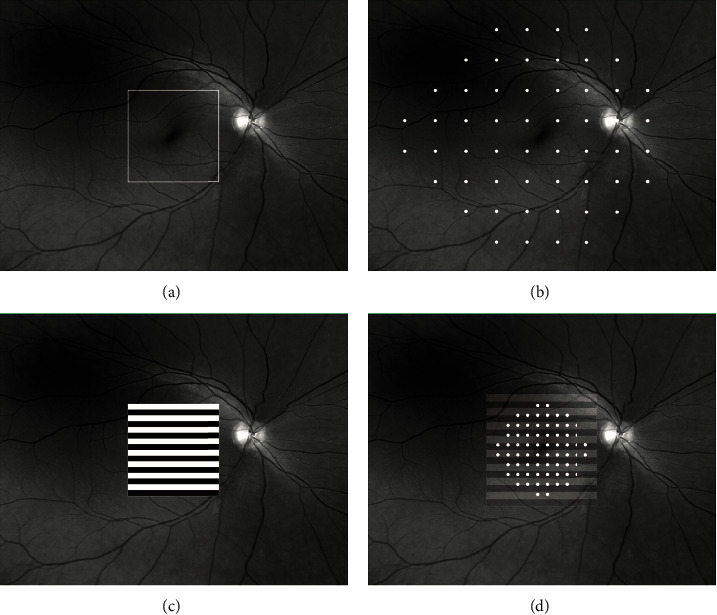
Schematic illustration of areas on the retina tested by different modalities. (a) Macular square area scanned by SD OCT. (b) Retinal area tested by HFA 24-2 and the demarcation of 54 test points across peripheral retina (point density is 6 degrees). (c) Steady state pattern electroretinogram (ss-PERG) stimulus superimposed on fundus photograph on the macular region. (d) Representation of ss-PERG stimulus superimposed on the macular region which roughly corresponds to the area covered by Humphrey field analyzer 10−2 strategy. The 68 test points of HFA 10-2 are demarcated (point density is 2 degrees).

Recent studies have demonstrated RGC loss which averaged 10.2% over the entire retina in early glaucoma, and a 5 dB local perimetric loss in sensitivity was associated with 25–50% RGC loss [4]. Before such defects occur, some eyes pass through a stage of increased fluctuations in perimetric sensitivity and the development of definitive field defects was preceded by a localized minor disturbance in the area where the defects appeared subsequently [17, 40]. Therefore, it became of interest to develop a new objective functional test that could provide an assessment of living RGCs before cell loss occurs. In this study, we used the HFA 24-2 test as a screening tool, testing the central 48° with a retinal coverage of 24° nasally and 30° temporally. This allowed us to exclude participants with different types of visual field defects and enabled us to include only participants deemed to have normal HFA 24-2. We subsequently used HFA 10-2, testing the central 20° (10° from fixation point in all direction), with the purpose of matching its retinal coverage with the ss-PERG stimulus area, which is testing the central 24° (12° from fixation point) (Figure 3). There is compelling structural and functional evidence that glaucomatous damage to the macula occurs in early stages of glaucoma [41]. However, glaucomatous damage to the macula is often missed in clinical practice when only 24-2 visual fields and peripapillary RNFL are used [42]. De Moraes et al. found that the 24-2 visual field test missed central damage detected with 10-2 tests in patients with ocular hypertension (OHTN), GS, and early glaucoma patients [43]. They reported that 10-2 tests revealed macular damage missed by 24-2 tests in OHTN, GS, and early glaucoma in 35, 39, and 61% cases, respectively [43]. When SD-OCT was used to detect the presence of macular damage and was used simultaneously with 10-2 tests, 24-2 tests still missed a significant number of eyes with macular damage (52%) [43].

A previous study compared VF defects and PERG parameters and found that deep defects in VF were associated with abnormal PERG [2]. Another study reported reduced PERG responses when scotomas were selectively stimulated [44]. These studies concluded that abnormal SAP and PERG measures represented RGC dysfunction; however, no studies studied this relationship in GS patients.

In our study, we demonstrated a significant positive correlation between all ss-PERG parameters and HFA indices, especially between MagD/Mag ratio and 24-2 VF MD (*r* > 0.390, *p* < 0.012), and 10-2 VF MD (*r* > 0.452, *p* < 0.003). These results reinforce the importance of using ss-PERG and HFA 10-2 when there is any evidence/concern about central damage and demonstrates the advantage of HFA 10-2 over 24-2 in GS.

Furthermore, decreased 10-2 VF MD represents an index of global functional depression and we reported a strong association between 10-2 VF MD and ss-PERG parameters, suggesting that the global functional depression was associated with RGC dysfunction (decreased ss-PERG parameters) in the central 20°. Decreased PSD is an index of focal functional loss, and we found that MagD/Mag ratio trended with 24-2 VF PSD (*r* = −0.286, *p*=0.07), suggesting nascent localized functional loss occurring close to Bjerrum's area. Visual field index (VFI) is a measure of RGC function, and VFI was significantly correlated with ss-PERG parameters. The lower the VFI of HFA tests, the more significant the RGC dysfunction. Based on these findings, we hypothesize that in GS subjects, the disease process begins with global functional depression in the central area, and it could progress onto future localized defects later.

We demonstrated a stronger relationship between ss-PERG parameters with 10-2 VF MD, when compared to 24-2 VF MD. These results are in line with a previous study that demonstrated HFA 24-2 testing strategy has limitations in early glaucoma, missing between 12% and 34% of eyes with confirmed glaucomatous macular damage [42]. The authors recommend the use of HFA 10-2 test or a modified 24-2 test instead, especially in patients with HFA 24-2 VF MD greater than −6 dB [42].

The HFA 24-2 strategies test employs a grid of 54 test locations evenly distributed with 6 degrees of separation between the locations. Only 12 of the 54 test point locations are in the central 10 degrees, while only 4 out of 12 test locations cover the central 8 degrees area, an area known to contain 30% of RGCs. Therefore, the central area populated by 30% of RGCs is functionally tested by only 4 test locations when the 24-2 SITA strategy is used. It is believed that this limitation is due to the low spatial resolution of this program in the macular region, leading to an underestimation of the functional deterioration in glaucoma, independent of the stage of the disease.

As demonstrated in this study, PERG is also often abnormal when the visual field tests show no defects in ocular hypertension [45] and early manifest glaucoma [46]. This dissociation between PERG and perimetry results in early stages of glaucoma is often found and is mostly because they test different aspects of the visual function. PERG is an objective measure of the electrical response of the central 40% of RGCs to a suprathreshold stimulus, while the perimetry is a subjective response to focal threshold stimuli covering central (10-2) and more peripheral retinal regions (24-2 or 30-2). Perimetry is believed to include the effects of RGC function as well as postretinal neural structures (lateral geniculate nucleus, cortex) which are subject to glaucomatous deafferentation [47, 48] and brain plasticity [49]. During aging, plasticity is essential for the normal adjustment of the brain to modifications in the sensory environment and plays an important role in recovery from damage to the visual system by compensating for gaps in perception, caused by glaucomatous deafferentation [50]. Cortical reorganization, with resulting filling-in, affects the early recognition of visual field defects [49] and may exacerbate the reduction of sensitivity due to RGC loss or mask it [46], causing affected subjects to ignore or underestimate their defects in the visual field [49]. Therefore, an abnormal PERG can be used to predict future losses of the visual field [51, 52], and PERG parameters precede the clinical signs of glaucomatous damage by a considerable period of time [51].

What about a normal PERG result despite an abnormal visual field test? These issues mostly occur in the presence of peripheral focal field losses, and we used 24-2 VF to exclude this scenario. Hood et al. [53] cautioned that the transient state PERG could miss glaucomatous damage in about 30% of patients with VF loss confirmed by multifocal visual evoked potentials. In contrast, we report no false negative test results utilizing ss-PERG. Another appealing explanation for normal PERG in patients with VF defects was offered by Caprioli et al. [54], suggesting that focal and diffuse VF loss may be caused by different mechanisms of glaucomatous optic nerve damage. When the VF loss is diffuse, the authors hypothesized that the process is IOP-dependent and is secondary to diffuse axonal dysfunction, leading to evenly distributed thinning of the disc rim. In these cases, PERG was abnormal and reflected diffuse RGC dysfunction [55]. In contrast, a localized VF loss is less IOP-dependent, and the accelerated RGC loss may be due to vascular and other neurotoxic factors, leading to scotoma formation. The number of affected RGCs must be substantial to reduce PERG parameters. In our study, we have also demonstrated a significant association between ss-PERG parameter (MagD/Mag ratio) and structural damage in the average and sectorial GCL + IPL thicknesses (Tables 4 and 5) and no contributions from 10-2 VF MD nor 24-2 VF MD, suggesting the importance of PERG testing in the early detection of glaucoma. Importantly, results from GLMM demonstrate highly significant correlation coefficients between MagD/Mag ratio and GCL + IPL thickness, and these coefficients were highly similar among average thickness and all sectoral thicknesses (*R* = 0.643–0.727) (Tables 4 and 5). Therefore, these findings suggest that ssPERG could detect RGC losses in a similar capacity caused by any of the abovestated mechanisms, regardless of a localized or generalized pattern of distribution.

A study examining average GCL + IPL thickness among glaucoma stages 1, 2, 3, and control group found thickness values to be 76.79 ± 8.05, 65.90 ± 7.92, 57.38 ± 10.00, and 86.01 ± 3.68 *μ*m, respectively [56]. Another study reported the average GCL + IPL thickness to be 81.10 ± 1.0 *µ*m for healthy controls and a decreased thickness to 66.50 ± 1.3 *µ*m in glaucoma patients [57]. In our study, average GCL + IPL thickness was 79.19 ± 9.62 *µ*m (Table 1), which suggested early structural damage while OCT might still indicate the “results being in the green.” It is believed that these findings are due to RGC synaptic dysfunction and the thinning of the RGC synaptic network.

In clinical practice when HFA and IOP are within normal values, RGC dysfunction could be the key in providing an objective assessment for patients at risk of converting from GS to overt glaucoma [58]. Our findings suggested that ss-PERG could be a useful tool for detecting early RGC dysfunction, especially when used with HFA 10-2 test and OCT imaging. In our study, we reported that some GS participants exhibited RGC dysfunction and these findings were consistent with previously published results and support the hypothesis that glaucoma is a progressive disease that begins with a “diffuse subclinical panretinal damage” of RGC which could be detected by electrophysiological testing by showing a decrease in magnitude (Mag) and/or an increase in latency (MagD and MagD/Mag ratio), while conventional SAP could not reflect such change in RGC function [2, 22, 59–61]. As the disease progresses, focal damage occurs and apparent scotomas will be found preferentially in the Bjerrum area. A sensitivity loss of 5 dB within 30° of the retina corresponded to a 20% loss of RGC, and the same loss of sensitivity within the central 10° was associated with 50% loss of RGC, but these results were not conclusive [2].

Electrophysiological tests have largely been relegated to large hospitals and research facilities due to their invasive and lengthy complex procedures, as well as difficulties associated with results' interpretation. New office-based devices are emerging in the market with simple and intuitive operator interfaces, standardized testing procedures, and simplified reports. Patient preparation has been simplified, and test duration has been reduced to approximately 2 minutes per eye. ss-PERG not only measures the strength of the electrical response (Mag), corresponding to the number of living RGC, but also detects the presence of ganglion cells in distress (MagD and MagD/Mag ratio). In most cases, the viability of these distressed cells indicates a synaptic dysfunction that is potentially reversible and could be fully restored with treatment [62]. ss-PERG can detect the onset of retinal dysfunction significantly earlier than the SAP [53]. Up to 75% of glaucoma goes undiagnosed due to the lack of adequate functional testing, and ss-PERG can provide the information needed in combination with eye examination and OCT imaging to diagnose glaucoma earlier.

This study had limitations. Race and ethnicity are significant risk factors for glaucoma, but it was not possible to access these data. Additionally, given our small population size of 25 patients, the power of our study was not as strong to detect a difference between our variables of interest. Future studies with larger population sizes must be conducted to investigate the use of ss-PERG in detecting RGC dysfunction in GS with normal HFA. Furthermore, we did not include axial length in our study; we used spherical equivalent instead, which is commonly used when axial length is not available. It is well documented in the literature the relationship between axial length and responses in flash ERG [63]. In particular patients who are myopic have been found to have longer peak times and reduced amplitude. It is also known that moderately myopic patients tend to have thinner peripapillary RNFL, mainly at the superior and inferior poles [64]. As we did not include patients' axial length, it is possible that myopia could have affected RNFL measurements. All subjects underwent HFA (24-2 and 10-2) testing on the same day. Even though subjects waited about 20 minutes between HFA testing, it is possible they were fatigued by the time they performed the second test, whether it was a HFA 24-2 or 10-2. Nevertheless, this effect was not biased towards one test more than the other, given that the HFA testing order (24-2 vs. 10-2) was randomly assigned. We concluded that ss-PERG testing had the ability to detect functional RGC damage in GS patients before it was discovered by HFA tests, and the ss-PERG parameter (MagD/Mag ratio) was found to be significantly associated with changes in average and sectorial GCL + IPL thicknesses, while HFA 24-2 and 10-2 tests were not associated. The relationship between PERG and HFA global indices has demonstrated that the glaucoma continuum began with a global deterioration of retinal function that later will lead to local VF defects. When ss-PERG detects functional deteriorations in nontreated patients, it would justify early intervention to treat functional alterations before irreversible structural damage occurs. The advantage of the ss-PERG in the hands of experienced clinicians could be used for improved monitoring of RGC dysfunction and its progression, after acquiring the necessary baselines. A normal 10-2 examination could also provide an important baseline for future comparison, especially when a central field deficit is suspected.

SAP often cannot detect significant differences between normal patients and GS patients due to its sensitivity [65]. Furthermore, results can be extremely variable, with patient focus and understanding playing significant roles in the validity of the measurements. Artifacts, such as cataracts or dry eye, can have significant impact as well [66, 67]. As a consequence, patients may progress further when earlier treatment could have been beneficial, with one study finding that almost half of all newly diagnosed glaucoma patients had an ophthalmic exam within a year prior, and less than one-fifth were diagnosed with glaucoma [68, 69]. This suggests that more sensitive tests are needed so that patients at risk of developing glaucoma can be treated earlier if deemed appropriate by the ophthalmologist. In this study, we demonstrated that ss-PERG parameters are associated with GCL + IPL thinning and are correlated with worse VF in GS patients. Thus, they could serve as an adjunct to SAP in clinical practice to help detect early changes in GS patients and allow for earlier interventions.

## 5. Consent

Written informed consent was obtained from all the subjects in this study.

## Figures and Tables

**Table 1:** Demographic and clinical characteristics of participants with preperimetric glaucoma.

*N* = 49 eyes (26 patients)	Mean ± SD
Age (years)	58.96 ± 12.82
Sex	16 females (62%)
BCVA (logMAR)	0.035 ± 0.084
IOP (mmHg)	17.43 ± 4.08
Vertical C/D ratio	0.63 ± 0.15

*Humphrey field analyzer*	
24-2 MD (dB)	−0.0004 ± 1.12
24-2 PSD (dB)	1.56 ± 0.43
24-2 VFI (%)	99.22 ± 0.90
10-2 MD (dB)	0.0361 ± 0.85
10-2 PSD (dB)	1.18 ± 0.20

*ss-PERG*	
Magnitude (*μ*V)	1.70 ± 0.63
MagnitudeD (*μ*V)	1.41 ± 0.68
MagnitudeD/Magnitude ratio	0.81 ± 0.13
Signal to noise ratio	5.34 ± 3.36

*GCL* *+* *IPL thickness in OCT*	
Superior sector (*µ*m)	77.45 ± 7.11
Superior nasal sector (*µ*m)	80.02 ± 9.33
Inferior nasal sector (*µ*m)	79.45 ± 12.92
Inferior sector (*µ*m)	77.64 ± 15.37
Inferior temporal sector (*µ*m)	80.68 ± 12.24
Superior temporal sector (*µ*m)	77.55 ± 6.70
Average GCL + IPL thickness (*µ*m)	79.19 ± 9.62

BCVA: best corrected visual acuity, IOP: intraocular pressure, C/D: cup-to-disc, MD: mean deviation, PSD: pattern standard deviation, VFI: visual field index, ss-PERG: steady state pattern electroretinogram, OCT: optical coherence tomography, GCL + IPL: ganglion cell inner plexiform layer.

**Table 2:** Comparison of demographic and clinical characteristics between eyes with normal and abnormal ss-PERG.

	Group 1 (normal ss-PERG)	Group 2 (abnormal ss-PERG)
	*N* = 34 eyes	*N* = 15 eyes

Age (years)^*∗*^	55.97 ± 11.35	64.87 ± 13.82
No. of females (%)	22 (64.7%)	9 (60%)
BCVA (logMAR)	0.03 ± 0.09	0.04 ± 0.06
Spherical equivalent (diopter)	−0.95 ± 2.60	−1.51 ± 2.40
Central corneal thickness (*µ*m)	546.14 ± 34.35	560.77 ± 26.20
Intraocular pressure (mm Hg)	17.29 ± 3.82	17.93 ± 4.88

24-2 VF MD (dB)^*∗*^	0.35 ± 0.92	−0.66 ± 1.06
24-2 VF PSD (dB)^*∗*^	1.44 ± 0.23	1.83 ± 0.64
VFI (%)^*∗*^	99.50 ± 0.66	98.64 ± 1.08
10-2 VF MD (dB)	0.21 ± 0.82	−025 ± 0.83
10-2 VF PSD (dB)	1.17 ± 0.21	1.22 ± 0.20
Rim area (mm^2^)^*∗*^	1.18 ± 0.17	1.02 ± 0.15

Disc area (mm^2^)	2.03 ± 0.56	1.81 ± 0.34
Average C/D ratio	0.64 ± 0.17	0.68 ± 0.15
Vertical C/D ratio	0.62 ± 0.16	0.66 ± 0.14
Cup volume (mm^2^)	0.33 ± 0.25	0.27 ± 0.16

*Circumpapillary RNFL thickness on spectral-domain OCT*		
Global average (*µ*m)^*∗*^	92.75 ± 9.04	85.50 ± 9.66
Superior quadrant (*µ*m)^*∗*^	110.22 ± 17.58	95.07 ± 13.46
Temporal quadrant (*µ*m)	68.53 ± 12.07	66.64 ± 20.31
Inferior quadrant (*µ*m)^*∗*^	122.12 ± 12.38	109.64 ± 15.79
Nasal quadrant (*µ*m)	70.38 ± 8.00	69.64 ± 9.55

*Macular GCL* *+* *IPL thickness on spectral-domain OCT*		
Average (*µ*m)	80.48 ± 5.48	76.92 ± 15.80
Minimum (*µ*m)^*∗*^	78.61 ± 5.45	71.54 ± 5.17
Superior sector (*µ*m)^*∗*^	79.97 ± 6.02	72.15 ± 5.65
Superior nasal sector (*µ*m)	81.24 ± 6.55	78.08 ± 13.87
Inferior nasal sector (*µ*m)	79.82 ± 6.04	79.62 ± 22.91
Inferior sector (*µ*m)	77.72 ± 5.88	78.54 ± 28.19
Inferior temporal sector (*µ*m)	81.00 ± 5.94	80.46 ± 21.79
Superior temporal (*µ*m)^*∗*^	79.18 ± 5.81	74.15 ± 7.38

Unless noted, values are expressed as the mean ± SD. ^*∗*^Significant difference between the two groups (*p* < 0.05). BCVA: best corrected visual acuity, MD: mean deviation, PSD: pattern standard deviation, VFI: visual field index, ss-PERG: steady state pattern electroretinogram, OCT: optical coherence tomography, RNFL: retinal nerve fiber layer, GCL + IPL: ganglion cell inner plexiform layer.

**Table 3:** Correlation analysis between ss-PERG and Humphrey field analyzer parameters.

	24-2 MD	24-2 PSD	24-2 VFI	10-2 MD	10-2 PSD
*Bivariate correlation analysis between ss-PERG and Humphrey analyzer parameters*					
Mag	*r* = 0.391^*∗*^	*r* = −0.249	*r* = 0.268	*r* = 0.450^*∗*^	*r* = −0.007
MagD	*r* = 0.522^*∗∗*^	*r* = −0.317^*∗*^	*r* = 0.387^*∗*^	*r* = 0.450^*∗*^	*r* = −0.052
MagD/Mag ratio	*r* = 0.425^*∗*^	*r* = −0.357^*∗*^	*r* = 0.473^*∗∗*^	*r* = 0.380^*∗*^	*r* = −0.190

*Partial correlation analysis between ss-PERG and Humphrey field analyzer parameters*					
Mag	*r* = 0.556^*∗∗*^	*r* = −0.222	*r* = 0.419^*∗*^	*r* = 0.563^*∗∗*^	*r* = −0.152
MagD	*r* = 0.610^*∗∗*^	*r* = −0.262	*r* = 0.500^*∗∗*^	*r* = 0.550^*∗∗*^	*r* = −0.159
MagD/Mag ratio	*r* = 0.390^*∗*^	*r* = −0.286	*r* = 0.470^*∗*^	*r* = 0.452^*∗*^	*r* = −0.237

ss-PERG: steady-state pattern electroretinogram, MD: mean deviation, PSD: pattern standard deviation, VFI: visual field index, Mag: magnitude, MagD: magnitudeD, MagD/Mag ratio: MagnitudeD/Magnitude ratio. ss-PERG parameters were transformed to achieve a normal distribution. ^*∗*^*p*  < 0.001; ^*∗∗*^*p*  <  0.05

**Table 4:** Generalized linear mixed models (unstructured covariance with random intercept) among ss-PERG and OCT parameters.

Average GCL + IPL					
	Coefficient	SE	F	Random effect	*p* value

Intercept	95.477	9.320	—	9.484	<0.001
Age (years)	−0.233	0.091	6.521	—	0.020
Spherical equivalent	1.092	3.840	10.337	—	0.023
IOP (mmHg)	−0.550	0.339	6.614	—	0.004
MagD/Mag ratio	9.397	0.214	5.987	—	0.017

Model summary	R (95% CI)		*F*		*p* value

	0.690 (0.483–0.825)		6.374		0.002

GCL + IPL: ganglion cell layer and inner plexiform layer, SE: standard error, IOP: intraocular pressure, MagD/Mag ratio: MagnitudeD/Magnitude ratio.

**Table 5:** GLMM analysis of MagD/Mag ratio with macular sectorial GCL + IPL thickness, controlling for age, IOP, and spherical equivalent.

Sectorial GCL + IPL	Random effect	*R* (95% CI)	*F*	*p* value
S sector	13.577	0.695 (0.509–0.818)	7.537	<0.001
SN sector	19.579	0.643 (0.437–0.785)	7.200	<0.001
IN sector	11.088	0.721 (0.547–0.835)	8.908	<0.001
I sector	11.234	0.727 (0.556–0.839)	7.737	<0.001
IT sector	11.711	0.651 (0.448–0.791)	7.610	<0.001
ST sector	11.888	0.663 (0.423–0.779)	5.991	0.003

GCL + IPL: ganglion cell-inner plexiform layer, IOP: intraocular pressure, MagD/Mag ratio: MagnitudeD/Magnitude ratio, S–superior, SN: superior nasal, IN: inferior nasal, I: inferior, IT: inferior temporal, ST: superior temporal.

## Data Availability

Readers can access the data analyzed in this manuscript by contacting the manuscript authors directly, as data are within SPSS and password protected due to PHI. Subjects are listed as numbers to ensure anonymity.

## References

[1] Ahmad S. S. (2018). Glaucoma suspects: a practical approach. *Taiwan Journal of Ophthalmology*.

[2] Bach M., Sulimma F., Gerling J. (1997). Little correlation of the pattern electroretinogram (PERG) and visual field measures in early glaucoma. *Documenta Ophthalmologica*.

[3] Quigley H. A., Dunkelberger G. R., Green W. R. (1989). Retinal ganglion cell atrophy correlated with automated perimetry in human eyes with glaucoma. *American Journal of Ophthalmology*.

[4] Kerrigan-Baumrind L. A., Quigley H. A., Pease M. E., Kerrigan D. F., Mitchell R. S. (2000). Number of ganglion cells in glaucoma eyes compared with threshold visual field tests in the same persons. *Investigative Ophthalmology & Visual Science*.

[5] Sommer A., Katz J., Quigley H. A. (1991). Clinically detectable nerve fiber atrophy precedes the onset of glaucomatous field loss. *Archives of Ophthalmology*.

[6] Haymes S. A., Leblanc R. P., Nicolela M. T., Chiasson L. A., Chauhan B. C. (2007). Risk of falls and motor vehicle collisions in glaucoma. *Investigative Opthalmology & Visual Science*.

[7] Chauhan B. C., Garway-Heath D. F., Goñi F. J. (2008). Practical recommendations for measuring rates of visual field change in glaucoma. *British Journal of Ophthalmology*.

[8] Maffei L., Fiorentini A. (1982). Electroretinographic responses to alternating gratings in the cat. *Experimental Brain Research*.

[9] Berardi N., Domenici L., Gravina A., Maffei L. (1990). Pattern ERG in rats following section of the optic nerve. *Experimental Brain Research*.

[10] Maffei L., Fiorentini A., Bisti S., Holländer H. (1985). Pattern ERG in the monkey after section of the optic nerve. *Experimental Brain Research*.

[11] Mathers K., Rosdahl J. A., Asrani S. (2014). Correlation of macular thickness with visual fields in glaucoma patients and suspects. *Journal of Glaucoma*.

[12] Banitt M. R., Ventura L. M., Feuer W. J. (2013). Progressive loss of retinal ganglion cell function precedes structural loss by several years in glaucoma suspects. *Investigative Opthalmology & Visual Science*.

[13] Bode S. F., Jehle T., Bach M. (2011). Pattern electroretinogram in glaucoma suspects: new findings from a longitudinal study. *Investigative Ophthalmology & Visual Science*.

[14] Bach M., Brigell M. G., Hawlina M. (2013). ISCEV standard for clinical pattern electroretinography (PERG): 2012 update. *Documenta Ophthalmologica*.

[15] Parisi V., Miglior S., Manni G., Centofanti M., Bucci M. G. (2006). Clinical ability of pattern electroretinograms and visual evoked potentials in detecting visual dysfunction in ocular hypertension and glaucoma. *Ophthalmology*.

[16] Porciatti V. (2015). Electrophysiological assessment of retinal ganglion cell function. *Experimental Eye Research*.

[17] Jafarzadehpour E., Radinmehr F., Pakravan M., Mirzajani A., Yazdani S. (2013). Pattern electroretinography in glaucoma suspects and early primary open angle glaucoma. *Journal of Ophthalmic and Vision Research*.

[18] Holder G. E. (2001). Pattern electroretinography (PERG) and an integrated approach to visual pathway diagnosis. *Progress in Retinal and Eye Research*.

[19] Porciatti V., Sorokac N., Buchser W. (2005). Habituation of retinal ganglion cell activity in response to steady state pattern visual stimuli in normal subjects. *Investigative Opthalmology & Visual Science*.

[20] Mavilio A., Sisto D., Ferreri P., Cardascia N., Alessio G. (2017). RE-PERG, a new procedure for electrophysiologic diagnosis of glaucoma that may improve PERG specificity. *Clinical Ophthalmology*.

[21] Porciatti V., Ventura L. M. (2004). Normative data for a user-friendly paradigm for pattern electroretinogram recording. *Ophthalmology*.

[22] Marx M. S., Bodis-Wollner I., Lustgarten J. S., Podos S. M. (1987). Electrophysiological evidence that early glaucoma affects foveal vision. *Documenta Ophthalmologica*.

[23] Tirsi A., Wong A., Zhu D., Stoffels G., Derr P., Tello M. C. (2022). Pattern electroretinogram parameters and their associations with optical coherence tomography in glaucoma suspects. *Journal of Current Glaucoma Practice*.

[24] Tirsi A., Gliagias V., Moehringer J. (2021). Pattern electroretinogram parameters are associated with optic nerve morphology in preperimetric glaucoma after adjusting for disc area. *Journal of Ophthalmology*.

[25] Tirsi A., Orshan D., Wong B. (2022). Associations between steady-state pattern electroretinography and estimated retinal ganglion cell count in glaucoma suspects. *Documenta Ophthalmologica*.

[26] Orshan D., Tirsi A., Sheha H. (2022). Structure-function models for estimating retinal ganglion cell count using steady-state pattern electroretinography and optical coherence tomography in glaucoma suspects and preperimetric glaucoma: an electrophysiological pilot study. *Documenta Ophthalmologica*.

[27] Brusini P., Filacorda S. (2006). Enhanced Glaucoma Staging System (GSS 2) for classifying functional damage in glaucoma. *Journal of Glaucoma*.

[28] Oli A., Joshi D. (2015). Can ganglion cell complex assessment on cirrus HD OCT aid in detection of early glaucoma?. *Saudi Journal of Ophthalmology*.

[29] Templeton G. F. (2011). A two-step approach for transforming continuous variables to normal: implications and recommendations for IS research. *Communications of the Association for Information Systems*.

[30] Chen M. J., Chang Y. F., Kuo Y. S., Hsu C. C., Ko Y. C., Liu C. J. (2019). Macular ganglion cell-inner plexiform vs retinal nerve fiber layer measurement to detect early glaucoma with superior or inferior hemifield defects. *Journal of the Chinese Medical Association*.

[31] McMonnies C. W. (2017). Glaucoma history and risk factors. *Journal of optometry*.

[32] Sommer A., Miller N. R., Pollack I., Maumenee A. E., George T. (1977). The nerve fiber layer in the diagnosis of glaucoma. *Archives of Ophthalmology*.

[33] Ventura L. M., Porciatti V. (2005). Restoration of retinal ganglion cell function in early glaucoma after intraocular pressure reduction: a pilot study. *Ophthalmology*.

[34] Crowston J. G., Fahy E. T., Fry L. (2017). Targeting retinal ganglion cell recovery. *Eye*.

[35] Schwartz M., Yoles E., Levin L. A. (1999). Axogenic and somagenic neurodegenerative diseases: definitions and therapeutic implications. *Molecular Medicine Today*.

[36] Zhou Y., Pernet V., Hauswirth W. W., Di Polo A. (2005). Activation of the extracellular signal-regulated kinase 1/2 pathway by AAV gene transfer protects retinal ganglion cells in glaucoma. *Molecular Therapy*.

[37] Morgan J. E., Datta A. V., Erichsen J. T., Albon J., Boulton M. E. (2006). Retinal ganglion cell remodelling in experimental glaucoma. *Advances in Experimental Medicine and Biology*.

[38] Weber A. J., Kaufman P. L., Hubbard W. C. (1998). Morphology of single ganglion cells in the glaucomatous primate retina. *Investigative Ophthalmology & Visual Science*.

[39] Morquette J. B., Di Polo A. (2008). Dendritic and synaptic protection: is it enough to save the retinal ganglion cell body and axon?. *Journal of Neuro-Ophthalmology*.

[40] Werner E. B., Drance S. M. (1977). Early visual field disturbances in glaucoma. *Archives of Ophthalmology*.

[41] Hood D. C., Raza A. S., de Moraes C. G., Liebmann J. M., Ritch R. (2013). Glaucomatous damage of the macula. *Progress in Retinal and Eye Research*.

[42] Grillo L. M., Wang D. L., Ramachandran R. (2016). The 24-2 visual field test misses central macular damage confirmed by the 10-2 visual field test and optical coherence tomography. *Translational Vision Science & Technology*.

[43] De Moraes C. G., Hood D. C., Thenappan A. (2017). 24-2 visual fields miss central defects shown on 10-2 tests in glaucoma suspects, ocular hypertensives, and early glaucoma. *Ophthalmology*.

[44] Marx R., Zrenner E. (1989). Sensitivity distribution in the central and midperipheral visual field determined by pattern electroretinography and harmonic analysis. *Documenta Ophthalmologica*.

[45] Porciatti V., Falsini B., Brunori S., Colotto A., Moretti G. (1987). Pattern electroretinogram as a function of spatial frequency in ocular hypertension and early glaucoma. *Documenta Ophthalmologica*.

[46] Bach M. (2001). Electrophysiological approaches for early detection of glaucoma. *European Journal of Ophthalmology*.

[47] Ventura L. M., Porciatti V. (2006). Pattern electroretinogram in glaucoma. *Current Opinion in Ophthalmology*.

[48] Yücel Y. H., Zhang Q., Weinreb R. N., Kaufman P. L., Gupta N. (2003). Effects of retinal ganglion cell loss on magno-parvo-koniocellular pathways in the lateral geniculate nucleus and visual cortex in glaucoma. *Progress in Retinal and Eye Research*.

[49] Safran A. B., Landis T. (1999). From cortical plasticity to unawareness of visual field defects. *Journal of Neuro-Ophthalmology*.

[50] Lam D. Y., Kaufman P. L., Gabelt B. T., To E. C., Matsubara J. A. (2003). Neurochemical correlates of cortical plasticity after unilateral elevated intraocular pressure in a primate model of glaucoma. *Investigative Opthalmology & Visual Science*.

[51] Pfeiffer N., Tillmon B., Bach M. (1993). Predictive value of the pattern electroretinogram in high-risk ocular hypertension. *Investigative Ophthalmology & Visual Science*.

[52] Bayer A. U., Erb C. (2002). Short wavelength automated perimetry, frequency doubling technology perimetry, and pattern electroretinography for prediction of progressive glaucomatous standard visual field defects. *Ophthalmology*.

[53] Hood D. C., Xu L., Thienprasiddhi P. (2005). The pattern electroretinogram in glaucoma patients with confirmed visual field deficits. *Investigative Opthalmology & Visual Science*.

[54] Caprioli J., Sears M., Miller J. M. (1987). Patterns of early visual field loss in open-angle glaucoma. *American Journal of Ophthalmology*.

[55] Ventura L. M., Porciatti V., Ishida K., Feuer W. J., Parrishii R. (2005). Pattern electroretinogram abnormality and glaucoma. *Ophthalmology*.

[56] Zivkovic M., Dayanir V., Zlatanovic M. (2018). Ganglion cell-inner plexiform layer thickness in different glaucoma stages measured by optical coherence tomography. *Ophthalmic Research*.

[57] Nouri-Mahdavi K., Nowroozizadeh S., Nassiri N. (2013). Macular ganglion cell/inner plexiform layer measurements by spectral domain optical coherence tomography for detection of early glaucoma and comparison to retinal nerve fiber layer measurements. *American Journal of Ophthalmology*.

[58] Bach M., Poloschek C. M. (2013). Electrophysiology and glaucoma: current status and future challenges. *Cell and Tissue Research*.

[59] Tsaousis K. T., Plainis S., Parry N. R., Pallikaris I. G., Tsilimbaris M. K., Detorakis E. T. (2013). Visual electrodiagnosis in glaucoma screening: a clinical study. *Journal of Glaucoma*.

[60] Langrová H., Jägle H., Zrenner E., Kurtenbach A. (2007). The multifocal pattern electroretinogram (mfPERG) and cone-isolating stimuli. *Visual Neuroscience*.

[61] Marmor M. F., Fulton A. B., Holder G. E., Miyake Y., Brigell M., Bach M. (2009). ISCEV Standard for full-field clinical electroretinography (2008 update). *Documenta Ophthalmologica*.

[62] Porciatti V., Ventura L. M. (2009). Physiologic significance of steady-state pattern electroretinogram losses in glaucoma: clues from simulation of abnormalities in normal subjects. *Journal of Glaucoma*.

[63] Grudzińska E., Modrzejewska M. (2021). Axial length in patients with myopia and interpretation of pattern electroretinogram recordings. *Clinical Ophthalmology*.

[64] Rauscher F. M., Sekhon N., Feuer W. J., Budenz D. L. (2009). Myopia affects retinal nerve fiber layer measurements as determined by optical coherence tomography. *Journal of Glaucoma*.

[65] Phu J., Khuu S. K., Yapp M., Assaad N., Hennessy M. P., Kalloniatis M. (2017). The value of visual field testing in the era of advanced imaging: clinical and psychophysical perspectives. *Clinical and Experimental Optometry*.

[66] Heuer D. K., Anderson D. R., Feuer W. J., Gressel M. G. (1989). The influence of decreased retinal illumination on automated perimetric threshold measurements. *American Journal of Ophthalmology*.

[67] Henson D. B., Emuh T. (2010). Monitoring vigilance during perimetry by using pupillography. *Investigative Ophthalmology & Visual Science*.

[68] Nayak B. K., Maskati Q. B., Parikh R. (2011). The unique problem of glaucoma: under‐diagnosis and over‐treatment. *Indian Journal of Ophthalmology*.

[69] Vijaya L., George R., Baskaran M. (2008). Prevalence of primary open-angle glaucoma in an urban south Indian population and comparison with a rural population. *Ophthalmology*.

